# Pharmacy use by dual-eligible non-elderly veterans with private healthcare insurance

**DOI:** 10.1186/s12913-016-1773-z

**Published:** 2016-09-23

**Authors:** Brian C. Lund, Mary E. Charlton, Alan N. West

**Affiliations:** 1 Center for Comprehensive Access & Delivery Research and Evaluation, Iowa City VA Health Care System (Mailstop 152), 601 Hwy 6 West, Iowa City, IA 52246 USA; 2 Department of Epidemiology, University of Iowa College of Public Health, 145 N. Riverside Drive, Iowa City, IA 52242 USA; 3 Veterans Rural Health Resource Center – Eastern Region, White River Junction VA Medical Center, White River Junction, VT 05009 USA; 4 Geisel School of Medicine, Dartmouth College, 1 Rope Ferry Rd, Hanover, NH 03755 USA

**Keywords:** Access to care, Veterans, Drugs, Health insurance

## Abstract

**Background:**

Utilization of private sector healthcare services among dual enrolled veterans with private healthcare insurance plans (PHIP) has not been well-characterized. Concurrent use of Veterans Health Administration (VHA) and non-VHA pharmacies may increase risk for adverse outcomes. Thus, the objectives of this study were to determine the extent to which dual VHA-PHIP enrollees obtain medications through VHA and non-VHA pharmacies and to characterize medications obtained through non-VHA pharmacies.

**Methods:**

This observational study used merged administrative data from VHA and a predominant regional PHIP to select veterans < 65 years of age, residing in two Midwestern US states, and simultaneously enrolled in both VHA and the PHIP during fiscal years (FY) 2001–2010. Primary outcome measures included counts of prescriptions dispensed from VHA and non-VHA pharmacies, and frequencies of medications dispensed by non-VHA pharmacies based on PHIP claims.

**Results:**

Of 5783 veterans who filled ≥ 1 prescription in FY10, 2935 (50.8 %) used non-VHA pharmacies exclusively, 1165 (20.2 %) used VHA pharmacies exclusively and 1683 (29.1 %) were dual users. Health services utilization was higher for dual users compared to exclusive users of either VHA or non-VHA pharmacies across multiple measures, including total prescriptions, outpatient encounters, and inpatient admissions. The most common medications dispensed by non-VHA pharmacies, by proportion of veterans treated, were hydrocodone (20.9 %), amoxicillin (18.5 %), simvastatin (17.5 %), azithromycin (17.4 %), and lisinopril (15.1 %). Antidepressants comprised 3 of 10 most common medications dispensed by VHA, but none of the most common medications dispensed to exclusive non-VHA pharmacy users.

**Conclusions:**

Our findings align with VHA-Medicare dual enrolled veterans where only a minority of veterans used VHA services exclusively. Younger veterans relied disproportionately on VHA for mental health medications.

## Background

The Veterans Health Administration (VHA) is the largest nationally integrated healthcare system in the United States, operating 168 medical centers and more than 1000 community based outpatient clinics. However, many veterans seek care outside the VHA system, particularly when they are also covered by state or federal programs such as Medicare or Medicaid, or have access to private health insurance, often through employment. Concerted efforts have been made to facilitate communication and coordinate care between VHA and private sector healthcare, but concurrent use of these systems adds to an already fragmented U.S health care system. The use of private sector healthcare services by veterans dual enrolled in Medicare and VHA has been well characterized in older adults [1–4]. Of dual enrolled elderly veterans who used outpatient healthcare services in 1999, 18 % were VHA only users, 36 % were Medicare only users, and the remaining 46 % were dual users of both VHA and private sector services [1]. A more recent report examining both inpatient and outpatient services during 2004–2009 for veterans dual enrolled in a Medicare Advantage plan found that 10 % of patients used VHA exclusively, 35 % used the Medicare plan exclusively, 50 % used both systems, and the remaining 4 % did not use services in either system [4]. In addition to potential inefficiencies and unnecessary duplication of services [4], a chief concern is that dual use may increase risk for adverse events arising from lack of coordination between systems, though studies supporting this hypothesis are inconclusive [5, 6].

While on-going examination of VHA-Medicare dual use in older adult veterans is warranted, virtually no data exist regarding younger veterans enrolled in a private health insurance plan (PHIP). The influx of younger veterans returning from service in Operation Enduring/Iraqi Freedom (OEF/OIF) makes this issue increasingly relevant, as most of these individuals will qualify for VHA enrollment, and many will concurrently have access to a PHIP through employment or purchased through Affordable Care Act exchanges. A large survey of veterans conducted in 1999 found that 32.7 % of VHA enrollees less than 65 years of age had private insurance without Medicare [3]. Of these, 16.8 % were VHA only users, 35.0 % used non-VHA services exclusively, 39.9 % were dual users, and the remaining 8.3 % used no services. Unfortunately, more recent data are unavailable and a more detailed characterization of the type of services used is also lacking. For example, in the Medicare aged population, the proportion of VHA patients who used Medicare-reimbursed services was approximately 30 % for primary care, exceeded 60 % for specialty services, but was only 3–4 % for mental health care [2].

One such service domain of particular salience is dual pharmacy use across systems, where lack of appropriate care coordination could lead to dangerous drug-drug interactions, as well as unnecessary duplication or gaps in therapeutic monitoring. One survey conducted among veterans age ≥ 65 years and engaged in care at a single VHA site in 2008 found that 44.3 % used only VHA pharmacies, and the remaining 55.7 % had some degree of non-VHA pharmacy use [7]. In this sample, 21.2 % of veterans had medication coverage through Medicare Part D, and a further 39.7 % of veterans had private insurance not obtained through Medicare Part D. Unsurprisingly, veterans with non-VHA drug benefits were significantly more likely to use non-VHA pharmacies. However, 43.8 % of veterans without any form of non-VHA drug coverage still reported some pharmacy use outside the VHA system. Unfortunately, no data are available concerning dual pharmacy use among younger veterans, pharmacy use among VHA enrolled veterans engaged exclusively in non-VHA care, or specific information about the most commonly filled medications outside VHA by dual users.

In order to address some of these important knowledge gaps, we acquired claims data from a predominant regional PHIP to combine service utilization data across both systems. Our study is the first to focus on pre-Medicare age veterans and examine how dually enrolled VHA-PHIP beneficiaries use VHA and non-VHA services. The specific objectives of this report are to determine the extent to which dual enrollees < 65 years obtain medications through VHA and non-VHA pharmacies, contrast patient characteristics among primary VHA pharmacy users, non-VHA pharmacy users, and dual users, and to characterize the most common medications obtained through non-VHA pharmacies.

## Methods

### Study population and cohort creation

Patients included all veterans under age 65 who resided in two Midwestern states and were simultaneously enrolled in both VHA and our partnered PHIP for at least one full fiscal year between October 1, 2000 and September 30, 2010. The current analysis focused on veterans who were dual enrolled during the most recent year (FY10). We also conducted some analyses among dual enrollees during FY02, the earliest year where pharmacy refill data was available, to examine potential changes in dual system pharmacy use over the study timeframe. The PHIP agreement stipulated that we could only link their claim-level data to aggregated, patient-level VHA data. Therefore, summary variables of VHA utilization were extracted by Veterans Affairs (VA) National Data Systems (NDS) for each fiscal year of enrollment and linked to person-level PHIP membership data extract. VHA summary variables included annual counts of: 1) inpatient medical admissions, 2) outpatient medical visits, 3) inpatient mental health admissions, 4) outpatient mental health visits, and 5) dispensed prescription medications. These variables were derived from the Medical SAS Outpatient Data and the Acute Care Medical SAS Inpatient Data constructed from the National Patient Care Database (NPCD), and the Decision Support System (DSS) Pharmacy Data. Comparable summary variables were created at the patient-level using claim-level PHIP data to measure private sector service utilization. Since VHA can bill PHIPs for services provided to veterans for non-service connected conditions, we identified all PHIP claims originating from VHA facilities based on their federal tax identification numbers, and these events were omitted from the PHIP utilization variables. Service connection is a proportion ranging from 0 to 100 % that is assigned by the U.S. Department of Affairs to individual veterans though a benefits review process and denotes the extent to which a veteran’s injury or disease is linked to their military service. Veterans with greater levels of service connection generally have lower out-of-pocket costs, such as reduced or eliminated copays for medications, which may impact their decision whether to use VHA versus non-VHA care.

### Pharmacy utilization

Patients were classified into pharmacy user status groups: exclusive VHA users, exclusive non-VHA users, or dual users. Individual prescription medications dispensed by non-VHA pharmacies were identified in PHIP claims using a text field containing the drug name. Medication frequencies included all single agent and combination products that contained that ingredient, identified using both brand and generic drug names. For example, the frequency of lisinopril use included all single agent drug products and combination products (e.g. lisinopril/hydrochlorothiazide combination products) and included all brand name versions of these products.

### Common VHA medications

Since we were restricted to patient-level aggregate VHA data, we could not identify individual medications dispensed by VHA to patients in our cohort. In order to provide a point of reference for interpreting the frequencies of common medications dispensed by non-VHA pharmacies, we created a proxy using the most common prescription medications dispensed to all VHA patients age 18–64 years receiving care at the 4 VHA medical centers with geographic boundaries overlapping the PHIP coverage area. These analyses used prescription data from the VA Decision Support System National Data Extract SAS Pharmacy datasets.

### Analysis

Differences in patient characteristics and health service utilization variables were compared across patient groups using chi-squared tests for categorical variables and analysis of variance for discrete variables. The equivalent nonparametric Wilcoxon rank sum test was substituted for variables that were not normally distributed. All statistical tests were conducted using SAS version 9.3 (Cary, NC) and were two-sided with a significance threshold of *α* = .05.

## Results

In FY02, 4022 non-elderly veterans were simultaneously enrolled in both VHA and the PHIP, which increased to 7087 veterans in FY10. Of the FY02 cohort, 1117 (27.8 %) patients were also dual enrolled in FY10. The mean age of dual eligible veterans increased from 51.2 to 52.4 years during this time period (Table 1). The distribution of age was highly skewed, where only 8.3 % of patients were < 35 years in FY02, and 40.8 % were 55–64 years. The over 55 age group increased to nearly 60 % of the sample by FY10. The majority of patients were male, though the proportion of women grew from 3.6 to 5.6 % during the observation period. The proportion of rural veterans decreased from 67.2 to 59.5 %.

**Table 1:** Patient characteristics

Characteristic	FY 2002	FY 2010	Statistics
	*N* = 4022	*N* = 7087	*X* ^2^ or Z; DF; p
Age in years, mean (S.D.)	51.2 (9.2)	52.4 (11.0)	*Z* ^a^ = 15.0; <.001
Age group, *n* (%)			*X* ^2^ = 362; 3; <.001
< 35 years	335 (8.3)	716 (10.1)	
35–44 years	490 (12.2)	963 (13.6)	
45–54 years	1556 (38.7)	1218 (17.2)	
55–64 years	1641 (40.8)	4190 (59.1)	
Sex, *n* (%)			*X* ^2^ = 22.4; 1; <.001
Male	3879 (96.4)	6693 (94.4)	
Female	143 (3.6)	394 (5.6)	
Marital Status, *n* (%)			*X* ^2^ = 48.6; 3; <.001
Married	1295 (32.2)	2677 (37.8)	
Never Married	144 (3.6)	294 (4.2)	
Divorced or Widowed	2323 (57.8)	3609 (50.9)	
Unknown	260 (6.5)	507 (7.2)	
Residence, *n* (%)			*X* ^2^ = 60.8; 1; <.001
Urban	1308 (32.5)	2819 (39.8)	
Rural	2702 (67.2)	4214 (59.5)	
Unknown	12 (0.3)	54 (0.8)	
Service Connection, *n* (%)			*X* ^2^ = 32.6; 4; <.001
None	2439 (60.6)	4408 (62.2)	
1–10 %	568 (14.1)	1079 (15.2)	
11–80 %	837 (20.8)	1397 (19.7)	
81–100 %	54 (1.3)	92 (1.3)	
Unknown	124 (3.1)	111 (1.6)	

^a^Wilcoxon rank sum test, normal approximation, two-sided test

**Fig. 1:**
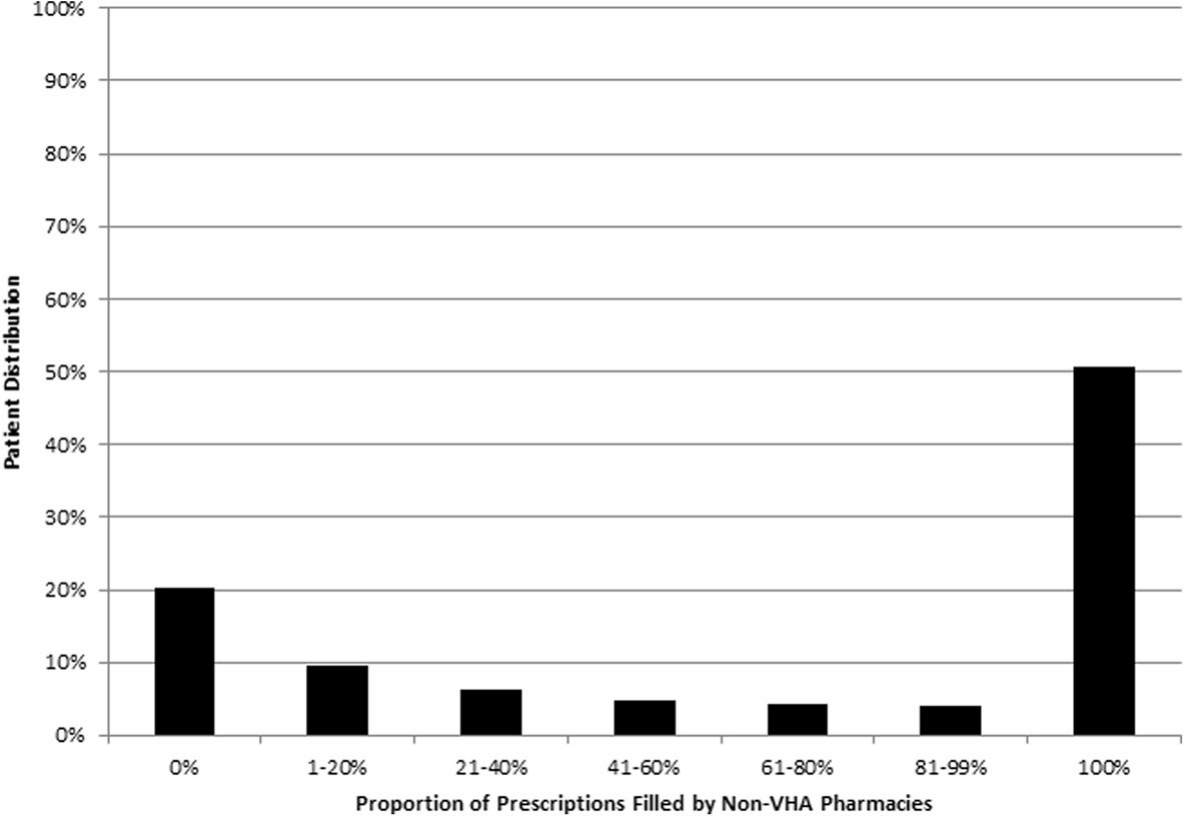
Proportion of Prescriptions Filled by Non-VHA Pharmacies in Non-Elderly, Dual-Eligible Veterans, 2010 (*N* = 5783)

Of the 7087 dual enrolled veterans in FY10, 5783 (81.6 %) filled at least one prescription. Of these, approximately half (*N* = 2935, 50.8 %) used non-VHA pharmacies exclusively, 20.2 % (*N* = 1165) used VHA pharmacies exclusively, and the remaining 29.1 % (*N* = 1683) had some degree of dual use between VHA and non-VHA pharmacies (Fig. 1). This distribution remained stable across individual years from FY02 through FY10, where the proportion of VHA only users varied from 18.6 to 22.1 % and non-VHA only users varied from 48.4 to 51.9 %.

Demographic characteristics and health service utilization were contrasted across FY10 pharmacy user status groups (Table 2). The proportion of women was highest among non-VHA only users (6.9 %) and lowest among VHA only users (3.4 %). The proportion of rural veterans was highest among dual users (64.2 %), compared to rates of 58.3 and 60.7 % for exclusive non-VHA and exclusive VHA users, respectively. The level of service connection was similar between VHA only and dual users, but significantly lower among non-VHA only pharmacy users. Overall health service utilization, which included both non-VHA care reimbursed by the PHIP and VHA care, was highest among dual users, including the number prescription fills, number of outpatient visits, and the proportion of patients who had an inpatient hospitalization. Dual users also had the highest mean number of outpatient mental health visits and the greatest proportion of patients with an inpatient mental health admission.

**Table 2:** Clinical characteristics of patients according to pharmacy user status, 2010

	Pharmacy user status group (*N* = 5783)			
Characteristic	VHA only	Dual	Non-VHA only	Statistics
	*N* = 1165	*N* = 1683	*N* = 2935	*X* ^2^ or F; DF; p
Age, mean (SD)	53.7 (10.2)	54.9 (9.7)	52.5 (10.8)	*F* = 29.2; DF = 2; *p* < .001
Sex, *n* (%)				
Male	1125 (96.6)	1590 (94.5)	2732 (93.1)	*X* ^2^ = 18.8; DF = 2; *p* < .001
Female	40 (3.4)	93 (5.5)	203 (6.9)	
Residence, *n* (%)				
Rural	707 (60.7)	1080 (64.2)	1712 (58.3)	*X* ^2^ = 15.7; DF = 4; *p* = .003
Urban	449 (38.5)	593 (35.2)	1197 (40.8)	
Unknown	9 (0.8)	10 (0.6)	26 (0.9)	
Service Connection, *n* (%)				
None	585 (50.2)	852 (50.6)	2070 (70.5)	*X* ^2^ = 447; DF = 8; *p* < .001
1–10 %	185 (15.9)	255 (15.2)	449 (15.3)	
11–80 %	369 (31.7)	523 (31.1)	331 (11.3)	
81–100 %	20 (1.7)	49 (2.9)	19 (0.7)	
Unknown	6 (0.5)	4 (0.2)	66 (2.3)	
Prescription fills, mean (SD)	17.6 (17.2)	31.7 (25.5)	21.7 (21.7)	*F* = 164; DF = 2; *p* < .001
Outpatient visits, mean (SD)				
Medical	14.3 (13.5)	23.6 (21.9)	13.6 (16.3)	*F* = 183; DF = 2; *p* < .001
Mental health	1.4 (4.1)	1.8 (5.5)	0.7 (5.6)	*F* = 26.9; DF = 2; *p* < .001
Total	15.7 (14.8)	25.4 (23.5)	14.2 (17.5)	*F* = 191; DF = 2; *p* < .001
Inpatient admission, *n* (%)				
Medical	46 (4.0)	177 (10.5)	214 (7.3)	*X* ^2^ = 43.1; DF = 2; *p* < .001
Mental health	5 (0.4)	16 (1.0)	5 (0.2)	*X* ^2^ = 14.6; DF = 2; *p* < .001
Any	50 (4.3)	191 (11.4)	219 (7.5)	*X* ^2^ = 48.8; DF = 2; *p* < .001

The medications most frequently dispensed by non-VHA pharmacies to dual enrolled veterans are listed in Table 3. Hydrocodone (with or without combined acetaminophen) was the most commonly filled medication and dispensed to 963 (20.9 %) unique patients. Five of the most common medications were antibiotics, including amoxicillin and azithromycin in the top 5, followed by cephalexin, ciprofloxacin, and levofloxacin. Several chronic medications for cardiovascular-related diseases were also present, including simvastatin and lisinopril in the top 5, as well as metoprolol, hydrochlorothiazide, metformin, and atorvastatin. The frequency of these non-VHA dispensed medications was further contrasted between dual pharmacy users and exclusive non-VHA pharmacy users. The consistent trend across medications was that frequencies for medications commonly taken on an acute basis did not differ between groups, whereas the frequency of chronic medications was significantly lower among dual users. For example, hydrocodone was dispensed to 963 (20.9 %) dual users and to 616 (21.0 %) non-VHA only users, which was not significantly different (*X*^2^ = .09; DF = 1; *p* = .766). In contrast, simvastatin was dispensed by non-VHA pharmacies to 180 (10.7 %) dual users versus 629 (21.4 %) non-VHA only users (*X*^2^ = 85; DF = 1; *p* < .001).

**Table 3:** Most common prescription medications filled in non-VHA pharmacies by dual enrolled patients, FY10

		Pharmacy user status		
Medication^a^	All patients *N* = 3773 *n* (%)	Dual users *N* = 1683 *n* (%)	Non-VHA only users *N* = 2935 *n* (%)	Statistics: dual vs. non-VHA only users *X* ^2^; DF; p
Hydrocodone	963 (20.9)	347 (20.6)	616 (21.0)	.09; 1; .766
Amoxicillin	853 (18.5)	338 (20.1)	515 (17.6)	4.6; 1; .033
Simvastatin	809 (17.5)	180 (10.7)	629 (21.4)	85; 1; <.001^b^
Azithromycin	802 (17.4)	304 (18.1)	498 (17.0)	.89; 1; .344
Lisinopril	699 (15.1)	185 (11.0)	514 (17.5)	35; 1; <.001^b^
Omeprazole/esomeprazole	477 (10.3)	127 (7.6)	350 (11.9)	22; 1; <.001^b^
Prednisone	429 (9.3)	173 (10.3)	256 (8.7)	3.1; 1; .079
Cephalexin	422 (9.1)	172 (10.2)	250 (8.5)	3.7; 1; .053
Metoprolol	391 (8.5)	105 (6.2)	286 (9.7)	17; 1; <.001^b^
Hydrochlorothiazide	374 (8.1)	92 (5.5)	282 (9.6)	25; 1; <.001^b^
Metformin	338 (7.3)	82 (4.9)	256 (8.7)	23; 1; <.001^b^
Ciprofloxacin	313 (6.8)	129 (7.7)	184 (6.3)	3.3; 1; .069
Levofloxacin	292 (6.3)	118 (7.0)	174 (5.9)	2.1; 1; .146
Cyclobenzaprine	289 (6.3)	88 (5.2)	201 (6.9)	4.8; 1; .029
Atorvastatin	243 (5.3)	41 (2.4)	202 (6.9)	42; 1; <.001^b^

^a^Includes all single agent and combination products that contain the ingredient medication (e.g. lisinopril includes combination formulations with other active ingredients such as hydrochlorothiazide; amoxicillin includes amoxicillin plus clavulanate combination products). Cell counts represent the number of unique patients who received each medication during FY10

^b^Statistically significant difference based on Bonferroni correction for multiple comparisons (*α* = 0.0033)

The frequency of the most common medications dispensed by VHA (i.e. the 4 VHA medical centers with geographic boundaries that overlapped the PHIP coverage areas) was contrasted with the most common medications dispensed to dual enrolled veterans who used non-VHA pharmacies exclusively (Table 4). Use of common chronic medications including lisinopril, metoprolol, metformin, and hydrochlorothiazide, was generally consistent between VHA patients and non-VHA pharmacy users in the older age group (45–64 years). Simvastatin use was more common in VHA, but comparable to the combined use of simvastatin plus atorvastatin use in the dual enrolled, non-VHA pharmacy user group. In contrast, important differences were observed in the younger age group (18–44 years), particularly related to psychotropic medication. The 10 most frequent medications in VHA included two antidepressants (citalopram and bupropion), one hypnotic (zolpidem), and one antidepressant used almost exclusively as a hypnotic (trazodone). No antidepressants appeared in the top 10 medications for the non-VHA pharmacy group.

**Table 4:** Most common medications dispensed in VHA contrasted with dual enrolled exclusive non-VHA pharmacy users, 2010

Age group: 18–44 years			
VHA system^a^	*N* = 10,371 *n* (%)	Dual enrollees who used non-VHA pharmacies exclusively	*N* = 694 *n* (%)
Es/omeprazole	1792 (17.3)	Hydrocodone	150 (21.6)
Hydrocodone	1766 (17.0)	Amoxicillin	140 (20.2)
Citalopram	1554 (15.0)	Azithromycin	134 (19.3)
Ibuprofen	1292 (12.5)	Prednisone	73 (10.5)
Simvastatin	1246 (12.0)	Cyclobenzaprine	72 (10.4)
Bupropion	1165 (11.2)	Cephalexin	55 (7.9)
Trazodone	1087 (10.5)	Es/omeprazole	44 (6.3)
Tramadol	1008 (9.7)	Simvastatin	42 (6.1)
Zolpidem	988 (9.5)	Lisinopril	40 (5.8)
Naproxen	952 (9.2)	Zolpidem	39 (5.6)
Age group: 45–64 years			
VHA system^a^	*N* = 37,795 *n* (%)	Dual enrollees who used non-VHA pharmacies exclusively	*N* = 2241 *n* (%)
Simvastatin	14,861 (39.2)	Simvastatin	587 (26.2)
Es/omeprazole	10,251 (27.0)	Lisinopril	474 (21.2)
Lisinopril	11,335 (29.9)	Hydrocodone	466 (20.8)
Hydrocodone	6960 (18.3)	Amoxicillin	375 (16.7)
Metoprolol	6048 (15.9)	Azithromycin	364 (16.2)
Vardenafil	5673 (15.0)	Es/omeprazole	306 (13.7)
Metformin	5670 (14.9)	Metoprolol	278 (12.4)
Hydrochlorothiazide	5519 (14.5)	Hydrochlorothiazide	267 (11.9)
Amlodipine	4348 (11.5)	Metformin	243 (10.8)
Albuterol inhaler	3615 (9.5)	Atorvastatin	196 (8.8)

^a^The VHA system included all patients receiving care at the 4 VHA medical centers with geographic borders that overlapped with the coverage area of the private health insurance plan. Cell counts represent the number of unique patients who received each medication during FY10

## Discussion

Our primary objective was to determine the extent to which younger, dual VHA-PHIP enrolled veterans obtained medications from VHA versus non-VHA pharmacies. We found that in FY10, approximately half (50.8 %) used non-VHA pharmacies exclusively, only 20.2 % used VHA pharmacies exclusively, and the remaining 29 % used both. This finding is similar to older, VHA-Medicare dual enrolled veterans, where 20 % or less of this population used VHA services exclusively [1, 4]. However, exclusive non-VHA pharmacy use was more common in our sample of younger PHIP enrolled veterans (50.8 %), compared to approximately 35 % in previous reports involving the older, Medicare enrolled veteran population [1, 4]. While the number of dual enrolled veterans increased, the distribution of VHA versus PHIP use was stable over the span of our observation period, which extended from FY02 to FY10. While not a focus of the current research, we hypothesize that the increase in dual enrollees resulted from increasing numbers of working age veterans enrolling in VA over the study period, and that use of VA health care has increased across all ages since 2005. Furthermore, the proportion of veterans under 35 years of age who are VA patients increased 3-fold during this period [8]. This growth has been attributed to increased outreach efforts by the VA, expanding lists of conditions that qualify for VA coverage, and a streamlined enrollment process [8].

Several differences in patient characteristics were observed across pharmacy user groups. Compared to the other two groups, exclusive non-VHA pharmacy users were disproportionately women and had lower rates of service connection. Dual users differed from both VHA and non-VHA exclusive users in having a higher proportion of rural residents and greater total service utilization across all domains, including inpatient hospitalizations, outpatient visits, and prescription fills. These patterns are generally consistent with previous reports involving dual VHA-Medicare users [1, 3, 9, 10]. VHA has made strides to address many of these issues, particularly in expanding women’s health services to meet the growing population of women veterans and in creating the VA Office of Rural Health in 2007 to expand access for rural veterans using telehealth and other innovative approaches to healthcare delivery. Future studies will be needed to determine the success of these initiatives in ensuring that all eligible veterans who want to receive their healthcare from VHA are able to access these services.

Our analysis further examined the specific medications most commonly dispensed to dual VHA-PHIP enrolled veterans in non-VHA pharmacies. The most common medications included treatments for both chronic diseases such as hyperlipidemia (simvastatin, atorvastatin), diabetes (metformin), and hypertension (lisinopril, metoprolol, hydrochlorothiazide), as well as acute indications such as infectious diseases (amoxicillin, azithromycin, cephalexin, ciprofloxacin, levofloxacin) and pain (hydrocodone). Among medications dispensed by non-VHA pharmacies, dual users and exclusive non-VHA pharmacy users had similar rates for acute medications, but dual users had significantly lower rates for chronic medications. This pattern suggests that both patient groups seek care outside VHA for acute conditions such as pain or infection at approximately equal rates. In contrast, dual users may be less likely to have chronic medications filled by non-VHA pharmacies because they are being filled and managed within VHA. Unfortunately, we could not test this assumption directly because we did not know which specific drugs were dispensed by VHA pharmacies, only the total number of prescriptions dispensed. Despite this important limitation, our findings suggest that dual users seek care across systems in a rational manner, selectively using VHA for the management of chronic disease states, but preferring more accessible non-VHA services for acute conditions.

In order to indirectly address the lack of drug specific information for prescriptions dispensed by VHA pharmacies to patients in our sample, we identified the most common medications dispensed to all veterans across the four VHA facilities that overlapped geographically with the PHIP catchment area as a proxy. The drugs most commonly prescribed to PHIP enrolled veterans who were exclusive users of non-VHA pharmacies was very similar to the VHA system as a whole for patients in the 45–64 year age range. Rates of use were nearly identical between systems for medications used to treat chronic diseases, including statins (simvastatin and atorvastatin), lisinopril, metoprolol, and others. These patterns suggest not only similar frequencies of common chronic diseases between VHA users and non-users in this age range, but also very similar provider choices regarding specific medications. However, prescribing patterns were substantially different for patients under 45 years. The antidepressants citalopram (15.0 %) and bupropion (11.2 %) were among the most common drugs in this age group in VHA. The antidepressant trazodone was also common (10.5 %), though this medication is used almost exclusively for insomnia. In contrast, the most common drugs dispensed to non-VHA pharmacy users did not include any antidepressants. This discrepancy has several potential explanations. First, prior reports have documented that veterans selectively seek mental health services from VHA [2]. This may be partly explained by individuals with major mental disorders having less access to employer-based PHIPs, or fewer mental health benefits offered by their PHIP than available through VHA. Alternatively, it is possible that PHIP patients more often choose non-drug treatments such as psychotherapy. Finally, rates of mental disorders may be similar across settings but the private sector is less adept than VHA in recognizing and screening their veteran patients for posttraumatic stress disorder, depression, suicidal ideation, and other related mental health issues.

This study is subject to several important limitations. First, we only had a count of prescriptions dispensed by VHA to our dual enrolled PHIP veterans but did not know specifically which medications were dispensed. We were thus unable to examine some important questions regarding dual VHA-PHIP pharmacy use, such as whether unsafe or duplicative medications were dispensed across systems to an individual patient. A further limitation was that we could only examine dual use in one regional Midwest PHIP. We did not have access to data from other PHIPs in the same region or information about out-of-pocket pharmacy purchases, and thus the actual rate of exclusive VHA pharmacy use may be even lower than our estimate of 20.2 %. Importantly, our PHIP accounts for 50–75 % of market share across the two states included in our study. We also do not know whether our findings can be generalized to other regions of the country, where factors such as regional differences in available PHIP benefits and VHA access could impact how dual enrolled veterans selectively use VHA versus non-VHA services. It is also unclear what impact recent legislation, such as the Affordable Care Act and the Veterans Access, Choice, and Accountability Act, may have on how dual enrolled patients seek services across systems.

## Conclusions

Our findings have important implications for various stakeholders. It is critical for national healthcare policy to understand how patients selectively interact with VHA and the private sector as beneficiaries of Medicare, Medicaid, PHIPs, and other payers. The consistency of our findings with prior studies of Medicare dual users suggests that patterns of dual use may be well-established prior to reaching Medicare eligibility. It is likely that PHIP enrolled veterans selectively use private sector services and then maintain this pattern as they transition to Medicare. Policy-makers and organizations specifically concerned with ensuring healthcare access among individuals with mental health disorders should recognize the key role that VHA plays for veterans with these illnesses. Our findings are relevant to VHA clinicians who face co-management across VHA and the private sector, and specifically with knowing common drugs their patients may be receiving from non-VHA sources and not necessarily well-documented in the VHA medical record. Our findings should also be of interest to investigators conducting research using VHA administrative data to understand what drug exposures are most likely to be missing from VHA data in pre-Medicare aged veterans and the potential impact on their findings. For example, studies concerned with identifying outpatient medication use for acute conditions such as pain or infection may be substantially biased since dual enrolled PHIP users frequently get these medications filled outside VHA. Our findings highlight the need for further research examining the potential risks, as well as potential benefits, of dual VHA and private sector healthcare use among younger veterans.
